# Investigation of Hybrid Nanoparticle–Acid Fluids (HNAFs): Influence of Wettability and Interfacial Tension Mechanisms in Harsh Carbonate Reservoirs for Improved Oil Recovery

**DOI:** 10.1021/acsomega.2c03626

**Published:** 2022-11-04

**Authors:** Mohamed Haroun, Md Motiur Rahman, Mohammed Al Kobaisi, Minkyun Kim, Abhijith Suboyin, Bharat Somra, Jassim Abubacker Ponnambathayil, Soham Punjabi

**Affiliations:** Khalifa University of Science and Technology, P.O. Box 127788, Abu Dhabi127788, UAE

## Abstract

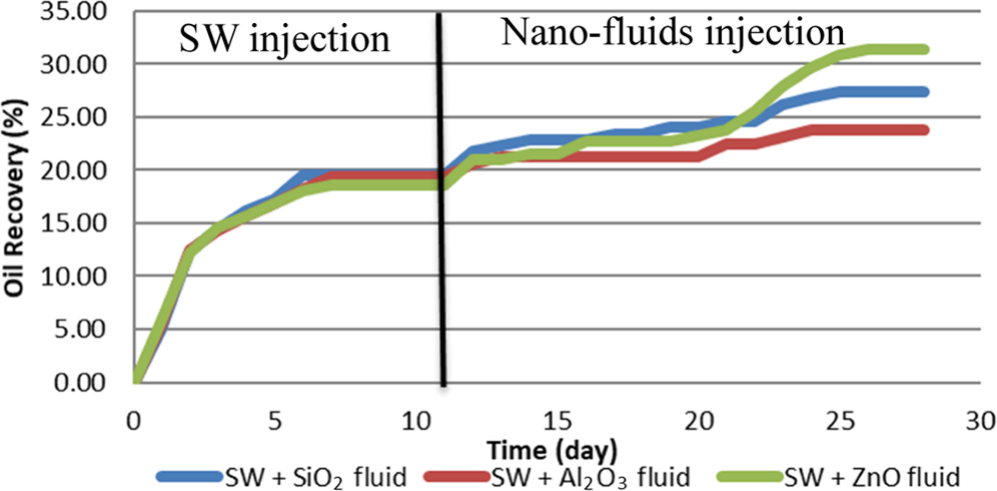

Over the past few years, there has been significant interest in the potential of hybrid nanoparticle–acid fluid (HNAFs) for improved oil recovery. This comprehensive study investigates the effects of nanoparticles and acid on interfacial tension (IFT) to establish a relationship between brine properties and the oil/brine IFT. This investigation is one of the first regional studies conducted utilizing candidate field data from the Middle East. Based on the literature review and screening studies conducted, a seawater (SW)-based HNAF was formulated with nanoparticles (SiO_2_, Al_2_O_3_, and ZnO) and HCl to measure their effect on IFT. A total of 48 formulations of HNAFs, nanofluids with and without acid, were analyzed with crude oil from a candidate field. IFT measurements were subsequently conducted using the pendant drop method under ambient conditions and in a high-pressure, high-temperature reservoir environment. Results showcased that IFT reduction was observed by increasing the acid concentration with SiO_2_ and Al_2_O_3_, although a reverse trend was observed with ZnO. Moreover, it was observed that IFT varied with increasing concentrations of nanoparticles, and at certain acid concentrations, IFT reduced significantly with higher nanoparticle concentrations. From the Amott studies, a clear signature was achieved, with ZnO exhibiting a total of 31.4% oil recovery, followed by SiO_2_ (27.3%) and Al_2_O_3_ (23.7%). The results of this study may assist in defining a screening criterion for future displacement (oil recovery) studies involving the presented nanoparticles. The results also reveal further the mechanisms involved in IFT reduction by hybrid nano–acid fluids and their potential for significant applications in the Middle East.

## 1 Introduction

Recently, nanoparticles have gained considerable interest due to their potential to modify thermal, electrical, and interfacial properties of crude oil in reservoirs. Nanoparticles (NPs) can enrich fluid–fluid properties, such as viscosity, interfacial tension (IFT), thermal conductivity, and fluid–rock properties, such as wettability and the heat-transfer coefficient, thus enhancing oil recovery.^1^

**Figure 1:**
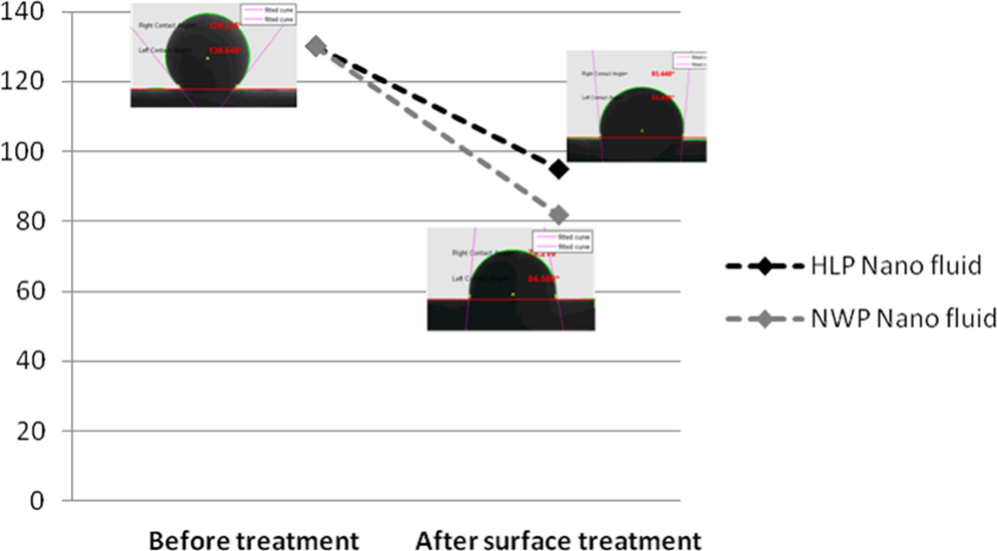
Comparison of the wettability conditions of sandstone slices with HLP and NWP.^4^

Polysilicon particles are a popular choice to alter the wettability and enhance oil recovery depending on the coating of the particle surface. Based on the wettability of the surface coating, polysilicons are classified into lipophobic and hydrophilic polysilicon (LHP), neutral wettable polysilicon (NWP), and hydrophobic and lipophilic polysilicon (HLP).^2^ Silicon dioxide, an LHP polysilicon, altered the wettability of sandstone surfaces from hydrophobic (oil-wet) to hydrophilic (water-wet) due to its adsorption/accumulation in pore throats.^3^ It was corroborated that the wettability alteration was relatively more influenced by the NWP type of nanoparticles than by HLP nanoparticles,^4^ as shown in Figure 1. Additional studies have also investigated the influence of wettability alteration by different concentrations of silica NPs, the variation in interfacial tension of solutions of surfactants and NPs, and the effect of the salinity of injected fluid on the contact angle, which result in a change in oil recovery. This included investigations on interactions of NPs with aqueous solutions in the pores of reservoirs and formation damage.^5−7^

**Figure 2:**
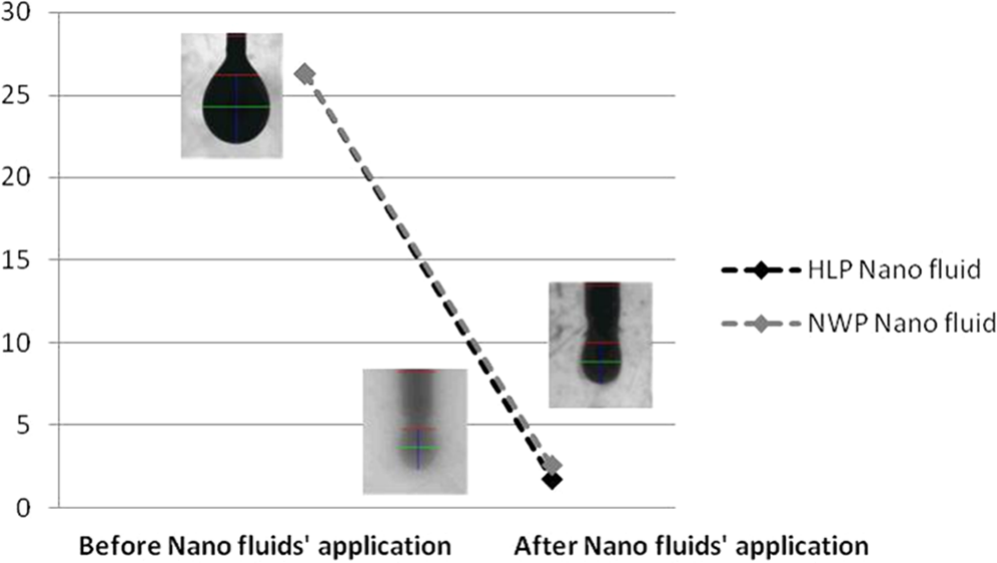
Comparison of the oil–water interfacial tension between HLP and NWP.^4^

Roustaei et al.^4^ also studied the effect of nanoparticles on the IFT between nanofluids (NWP and HLP) and crude oil. They observed that both NWP and HLP were effective in decreasing the IFT, as shown in Figure 2. Olayiwola et al.^8^ reported that the interaction of NPs, surfactants, and electrolytes at the solid–liquid interface depends on the surface charge of NPs and surfactants. They further indicated how dipole–dipole interactions of the ions, in addition to the electric double-layer effect and the cohesive energy, contribute to the stability of oil–water emulsions and the reduction in interfacial energy. The IFT reduction potential of nanofluids was confirmed by Suleimanov et al.,^9^ who observed that sulphanole solution decreased IFT by 70–90%. In addition, dielectric nanofluids, including ZnO and Al_2_O_3_, were highly effective in reducing the IFT.^10^ Alomair et al.^11^ showed that lower concentrations of nanofluids such as SiO_2_, Al_2_O_3_, TiO_2_, and NiO reduced the IFT, but higher concentrations resulted in a negligible reduction. Ravera et al.^12^ investigated how dispersed nanoparticles in an aqueous phase can modify the interfacial properties of liquid/air or liquid/liquid systems if their surface is modified by the presence of an ionic surfactant.

Previous studies were conducted on smart water and nanofluid flooding and low-concentration acidizing (LCA).^13−19^ Yuan et al.^20^ reported that when different concentrations (1, 0.1, and 0.01%) of liquid nanofluids (LNFs) are used in deionized water (DI), imbibition occurs due to the wettability alteration in oil-wet porous media. Furthermore, the rate of wettability alteration increases as the concentration of LNF is increased, and it was shown that 1% LNF solution had the best effect on the imbibition rate and wettability alteration. Neubauer et al.^21^ utilized two types of nanofluids, surface-modified silicon nanoparticles and a blend of solvent, surfactant, and surface-modified SiO_2_ nanoparticles in synthetic brine, to showcase that IFT reduction was observed in both nanofluids. The blend resulted in lower values, which might be due to the presence of the surfactant, and experiments indicated that wettability alteration can be achieved by both nanofluids. Afekare et al.^22^ also showed how aqueous dispersions of hydrophilic nanosilica may have a significant effect on the reduction (>70%) of the rock–oil adhesion force and work of adhesion (>95%), which leads to nanoscale wettability improvement and a potential increase in the recovery factor. Kaito et al.^23^ utilized a 0.5 wt % surface-modified nanosilica dispersion (SND), which contains 18% colloidal silica nanoparticles and some chemicals to enhance the stability of nanoparticles in synthetic brine, to show that nanoparticles increased oil recovery by 14 points in most core-flooding experiments.

Deng^24^ integrated nanoparticles with LCA and suggested the impact of hybrid nano–acid fluids (HNAF) on the fluid–fluid interaction by conducting IFT measurements. On the other hand, LCA combined with electrokinetics reduces interfacial tension, alters wettability, enhances the capillary number, and finally increases the displacement efficiency.^25^ CuO, NiO, SiO_2_, and Al_2_O_3_ nanoparticles added to HCl solution were categorized into two groups depending on the existence and nonexistence of surface charge. It was observed in all tested fluids that an increase in acid concentration resulted in IFT reduction, while higher concentrations of nanoparticles had no effect on reducing the IFT. In addition, compared with acid solutions without nanoparticles and seawater, the presence of nanoparticles decreased the IFT by 16.8 and 56%, respectively. Hybrid nano–acid fluids with Al_2_O_3_ and SiO_2_ showed a significant increase in oil recovery compared with seawater. Therefore, based on the literature and via IFT measurements, Al_2_O_3_ and SiO_2_ acid fluids were selected for further studies due to their stability and higher surface area. Results from previous investigations, even with smart brines, have also been incorporated to comprehend and expand the impact of HNAFs.^26,27^

## 2 Materials and Methods

In this study, systematic experimental analyses were conducted using representative materials (core plugs, crude oils, and formation water/seawater solutions). A reservoir temperature of 90 °C was maintained where possible. Materials and methods used are defined in the following sections.

### 2.1 Reservoir Rock Sample

Core plugs retrieved from the Indiana limestones outcrop with 99% calcite, representing Abu Dhabi carbonate reservoirs, were used for the spontaneous imbibition test. Conventional core analysis was performed on the received plugs, and their petrophysical properties were measured. The results are listed in Table 1.

**Table 1:** Core Properties Used for Spontaneous Imbibition

core plug ID	diameter (cm)	length (cm)	pore volume (cm^3^)	porosity (%)	*K*_liq_ (mD)	OOIP (cm^3^)	*S*_wi_ (%)
A-1	3.767	7.603	14.59	17.22	2.328	8.955	38.62
A-2	3.778	7.598	12.661	14.86	1.542	7.994	36.86
A-3	3.769	7.618	13.876	16.33	1.165	8.598	38.04

### 2.2 Crude Oil

Filtered crude oil retrieved from Abu Dhabi reservoirs was used to determine the IFT and aging of core plugs. The properties of crude oil are shown in Table 2. Each core plug was aged for 14 days.

**Table 2:** Density and Viscosity of the Oil at Each Temperature of Brines

temperature (°C)	20	25	50	75	90
density (g/cm^3^)	0.8232	0.8197	0.8023	0.7846	0.7753
viscosity (cP)	3.8414	3.3288	1.9637	1.2603	1.0615

Synthetic formation brines were used to saturate reservoir rock samples based on the ionic composition of the formation water retrieved from Abu Dhabi reservoirs. Abu Dhabi representative seawater was used as a base for the HNAF studied. The composition of the formation water and seawater tested are shown in Table 3.

**Table 3:** Ion Concentrations of Formation Water and Seawater

	Na^+^	Ca^2+^	Mg^2+^	K^+^	Cl^–^	SO_4_^2–^	HCO_3_^–^	TDS (ppm)
FW	44.261	13.84	1.604		96.5659	0.885	0.332	157.488
SW	19.054	0.69	2.132	0.672	30.924	3.944	0.123	57.539

### 2.3 Hybrid Nano–Acid Fluids (HNAFs)

In this study, Si0_2_, Al_2_O_3_, and ZnO nanoparticles, as mentioned in Table 4, were selected to prepare HNAFs with different concentrations (3–6 wt %) of hydrochloric acid to observe the best formulations. Forty-eight combinations of HNAFs, as indicated in Table 5, were prepared using the ultrasonicator equipment to form a nanoparticle suspension. Then, the dispersed nanoparticle solution was retrieved immediately out of the sonicator for the IFT measurement and Amott test. In addition, it was observed that the acid did aid in maintaining the uniform distribution of fluids. Acid solutions without nanoparticles and nanoparticle fluids without acid were tested as the baseline. Therefore, a total of 60 fluids were tested. Corrosion inhibitors were added to the HNAF solutions to preserve the equipment. The major criteria for the shortlisted brine include identifying an optimum concentration for the nanoparticles and acid while preventing corrosion.

**Table 4:** Nanoparticle Properties

products	SiO_2_	Al_2_O_3_	ZnO
form	powders	powders	powders
particle size (nm)	10–20	30–60	≤100
molecular weight (g/mol)	60.08	101.96	81.37
density (g/cm^3^)	2.2	3.27	5.61
pH	3.7–4.5	6–7	6–9
surface area (m^2^/s)	300	130	15–25
surface charge	positive	positive	positive

**Table 5:** Hybrid Nano–Acid Fluid Formulations

SiO_2_(wt %)	acid (wt %)	Al_2_O_3_(wt %)	acid (wt %)	ZnO (wt %)	acid (wt %)
0.1	3	0.01	3	0.4	3
0.2	4	0.04	4	0.6	4
0.3	5	0.07	5	0.8	5
0.4	6	0.1	6	1	6
4 × 4 = 16		4 × 4 = 16		4 × 4 = 16	
the number of scenarios = 48					

### 2.4 Experimental Procedure

The following subsections detail the experimental procedure for measuring IFT and oil recovery through spontaneous imbibition.

#### 2.4.1 Interfacial Tension

**Figure 3:**
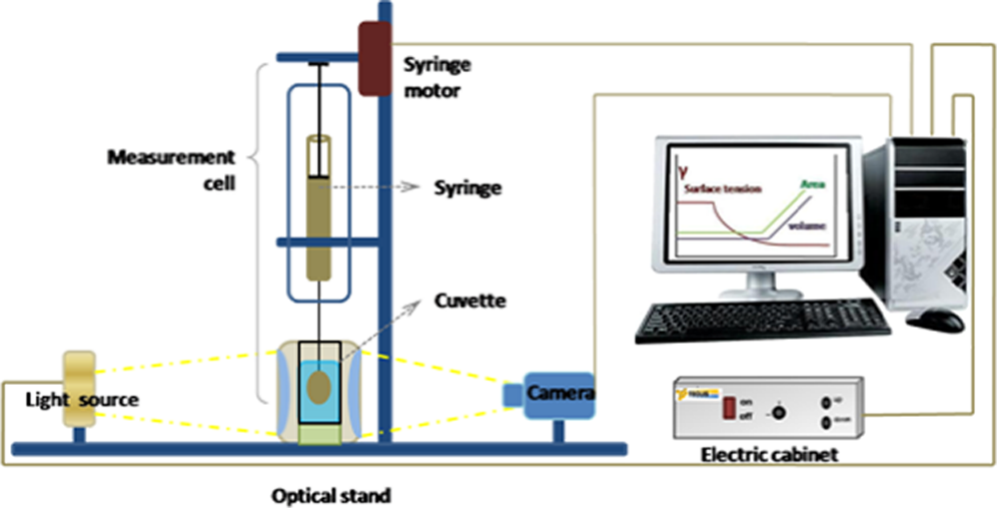
Schematic of IFT measurement (pendant drop system).

The pendant drop method was used to measure the interfacial tension (IFT) of HNAFs both under ambient conditions and under high-pressure and high-temperature (HPHT) conditions. Tracker software was used to capture and calculate the IFT based on the Young Laplace equation. The pendant drop system is shown in Figure 3.

The following steps were conducted:

1. A filling syringe was designed for the system with the oil sample and a cuvette with 25 mL of brine sample.
2. The density of oil and brine was input into the software before measuring IFT.
3. Measurement under ambient temperature and pressure conditions: the syringe was mounted tightly onto the device, and the cuvette was aligned with the camera. The dispenser needle was soaked into the cuvette and the tip of the needle was centered by adjusting the height and direction of the device.
4. Measurement at HPHT: the cuvette and syringe were placed into the chamber with a temperature probe and the cap was tightened. The prepared chamber was placed on the equipment under a pressure of 200 psi by connecting it to a nitrogen gas tank and the temperature was increased to 90 °C.
5. Measurements were performed on Tracker software, which captured the contour of the oil drop, calculated the interfacial tension value, and read the value every 1 min for 10 min until the reading stabilized. During drop formation, when the drop volume reaches a certain level, the speed of drop formation will decrease. The volume of the drop to be formed slowly reaches closer to the volume of the drop defined. The value of the initial drop-volume tolerance is defined as 1% in the system. Tracker uses the axisymmetric drop shape analysis technique to find the interfacial tension by fitting the Laplace equation.

IFTs between crude oil and various combinations of HNAFs were tested at ambient temperature. Three best brines from each category of nanoparticles were selected for further studies under HPHT conditions.

#### 2.4.2 Spontaneous Imbibition

**Figure 4:**
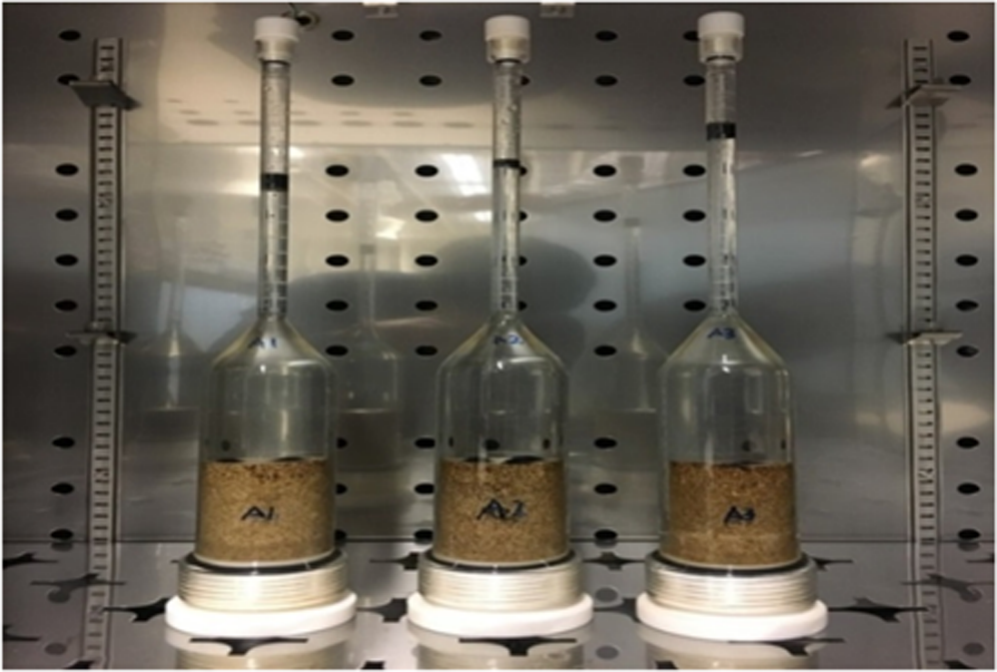
Setup for spontaneous imbibition test.

Oil displacement by spontaneous imbibition was measured using Amott cells at 90 °C, as shown in Figure 4. Core plugs were first subjected to seawater. Once the oil production plateaued, the brine was switched with nanofluids (without acid) to determine the incremental recovery indicating a wettability alteration.

The following steps were conducted:

1. The core plugs were taken out from the aging cell, excess oil on the surface was removed by rolling on nonabsorbent paper, and the weight was measured.
2. The core plugs were placed in Amott cells.
3. Degassed brines were siphoned into the Amott cell slowly to ensure that no air bubbles were introduced in the Amott cells and around the core.
4. The sequence of changing the brines was followed and the oil volume was recorded every 24 h.

## 3 Results and Discussion

The results obtained based on the systematic experimental procedure are discussed in the following sections to define a screening criterion for future oil recovery studies.

### 3.1 Interfacial Tension under Ambient Conditions

**Figure 5:**
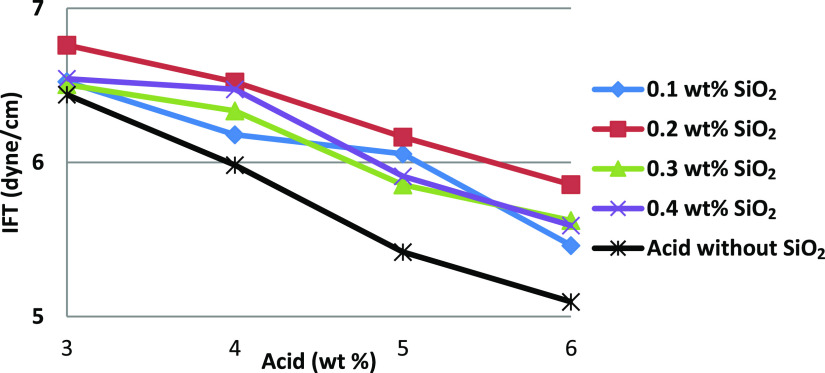
Interfacial tension of SiO_2_–acid fluids organized in terms of acid concentration.

**Figure 6:**
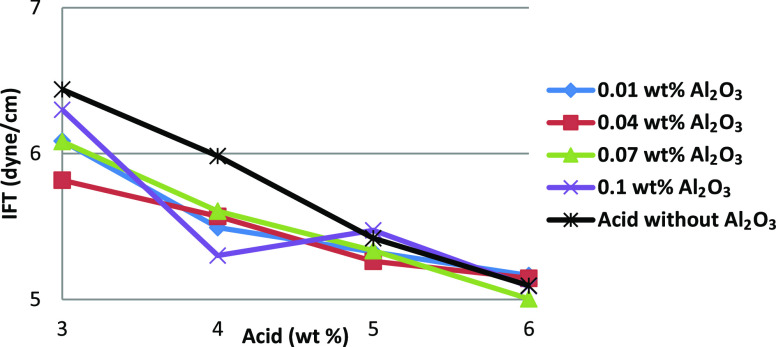
Interfacial tension of Al_2_O_3_–acid fluids organized in terms of acid concentration.

**Figure 7:**
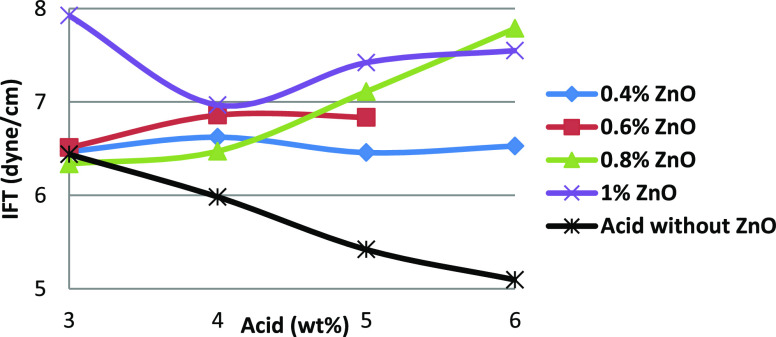
Interfacial tension of ZnO–acid fluids organized in terms of acid concentration.

The IFTs of 60 HNAF formulations with crude oil were studied in this phase of the research. The fluids were divided into three categories depending on the nanoparticles used, as shown in Table 5. The IFT of seawater under ambient conditions was found to be 15.16 dynes/cm. The dispersed nanoparticles in an aqueous phase usually modify the interfacial properties of liquid–liquid systems.^11^ As observed in Figures 5–7, dispersed nanoparticles in the acidic phase decreased the IFT by more than 50%. It was also observed that increasing the acid concentration resulted in a higher reduction in the IFT.

As seen in Figure 5, IFTs with various concentrations of SiO_2_ decreased with increasing acid concentration. However, the addition of nanoparticles had a lower effect on IFT reduction compared with acid.

Figure 6 shows the effect of Al_2_O_3_ with acid on the IFT between the HNAF fluid and crude oil. Compared with SiO_2_, the addition of Al_2_O_3_ showed a further reduction in IFT with respect to the acid concentration. Also, alumina acid fluids showed a lower IFT than SiO_2_ acid fluids at much lower concentrations. With a higher concentration of Al_2_O_3_ (0.1 wt %), the rate of acid reaction may be less during an acid concentration increase of 3–4% than that during an acid concentration increase of 4–5%. Therefore, during a 3–4% increase, the in situ live acid is higher and IFT is decreased. Similarly, during a 4–5% increase, the in situ live acid may be less and IFT is increased at an acid concentration of 5% and then IFT decreased again, which may be because of the acid reaction kinetics. However, the in situ live acid available on the face of the pore space and nanoparticles depends on the rate of the acid reaction (kinetics and equilibria features).

Figure 7 shows the IFT measurement of a ZnO-based HNAF. Although the ZnO–acid fluid showed a decrease in IFT compared with seawater, the addition of ZnO at higher acid concentrations increased the IFT compared with fluids without nanoparticles. Therefore, ZnO nanoparticles do not affect the IFT in the acidic aqueous phase.

### 3.2 Interfacial Tension under HTHP Conditions

**Figure 8:**
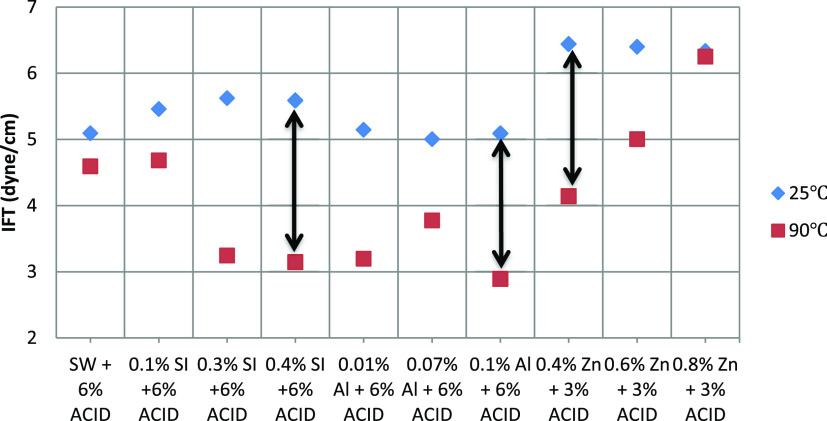
Comparison of nine IFT values obtained under ambient and HPHT conditions.

Based on the IFT results under ambient conditions, three fluids from each category that achieved low IFT values were selected to measure the IFT under HPHT conditions. The selected fluid formulations, along with the IFT under ambient and HPHT conditions, are presented in Table 6. As can be observed in Figure 8, the IFT at HPHT was lesser than that under ambient conditions. Therefore, the acidic aqueous phase and temperature have a direct effect on IFT reduction while Abu Dhabi reservoirs are at about 90 °C.

**Table 6:** IFT Values under Both Ambient and High-Pressure High-Temperature Conditions

fluid composition	20 °C(dyne/cm)	90 °C(dyne/cm)
0.1% SiO_2_ + 6% acid	5.5	4.7
0.3% SiO_2_ + 6% acid	5.6	3.2
0.4% SiO_2_ + 6% acid	5.6	3.1
0.01% Al_2_O_3_ + 6% acid	5.1	3.2
0.07% Al_2_O_3_ + 6% acid	5.0	3.8
0.1% Al_2_O_3_ + 6% acid	5.1	2.9
0.4% ZnO + 3% acid	6.4	4.1
0.6% ZnO + 3% acid	6.4	5.0
0.8% ZnO + 3% acid	6.3	6.3

### 3.3 Spontaneous Imbibition

The impact of nanoparticles on wettability alteration was studied using spontaneous imbibition at 90 °C without the addition of acid for this phase of the study. To prevent precipitation, the solutions were sonicated and heated to 90 °C before being poured into the Amott cells. Seawater was used as the base brine for secondary recovery (without any acid) before subjecting the core plug to the nanofluid.

**Figure 9:**
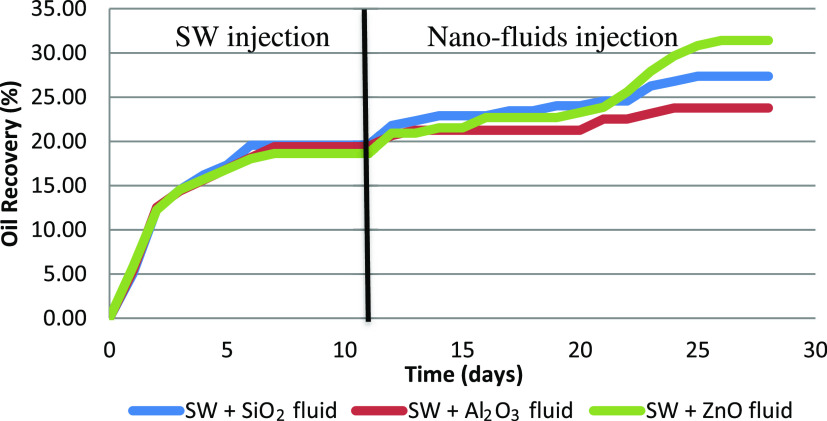
Oil recovery by all three tested hybrid nanofluids by the Amott test (90 °C).

Figure 9 demonstrates the incremental oil recovery for the three tested hybrid nanofluids while using SiO_2_ fluid (0.4% silica dioxide), Al_2_O_3_ fluid (0.1% aluminum oxide), and ZnO fluid (0.4% zinc oxide), respectively, without acid. These nanoparticle concentrations yielded the best results in the IFT test. This study confirmed the wettability alteration capabilities of the tested nanofluids. During the first phase of the Amott test, all three base fluids exhibited the same behavior on wettability alteration across the three tested core plugs. However, during the second phase of the test, a clear trend was observed as of the 12th day, with the SiO_2_-based nanofluid outperforming the other two because the nature of SiO_2_ is not vulnerable to high-temperature and high-salinity conditions but stable without deformation compared with other nanoparticles. ZnO negatively affected the permeability of the plugs by blocking the pores, and Al_2_O_3_ has the ability to reduce oil viscosity. The ZnO-based nanofluid response was delayed for about 10 additional days, after which it outperformed the other two nanofluids, indicating a longer residence time requirement for Zn to complete the ionic exchange. At the end of the test, a clear signature was achieved, with the ZnO nanofluid achieving a total of 31.4% oil recovery, followed by SiO_2_ (27.3%) and Al_2_O_3_ (23.7%). The incremental recovery of each tested nanofluid was led by the ZnO nanofluid (12.79%), followed by SiO_2_ (7.82%) and Al_2_O_3_ (4.38%). The varying responses of each of the shortlisted nanoparticles is highly indicative of in situ reactions taking place that are a function of time, which need to be assessed in depth.

## 4 Conclusions

This investigation presents one of the first regional studies conducted utilizing candidate field data from the Middle East to analyze the influence of wettability and interfacial tension mechanisms in harsh carbonate reservoirs for improved oil recovery. Based on the nanoparticles (SiO_2_, Al_2_O_3_, and ZnO) used, there is an indication of wettability alteration along with the potential for additional oil recovery.

The following are the key conclusions deduced from this study:

1. Pendant drop experiments performed at both ambient temperature and 90 °C revealed that the IFT was reduced by around 2-fold with increased acid concentration compared with seawater with no acid. Under ambient conditions, it was revealed that the Al_2_O_3_-based hybrid nano–acid fluid at its lowest concentration outperformed the other hybrid nano–acid fluids across the tested concentrations (0.01–0.1 wt %).
2. At 90 °C, SiO_2_-based hybrid nano–acid fluids at their highest test concentrations resulted in an IFT reduction trend similar to that observed with the Al_2_O_3_-based hybrid nano–acid fluids.
3. In comparison, ZnO-based hybrid nano–acid fluids may have indicated in situ reactions consuming the acid, resulting in the weaker performance in IFT reduction.
4. All three nanoparticles (SiO_2_, Al_2_O_3_, and ZnO) dispersed in seawater were tested in this study, which indicated a significant effect on wettability alteration. They demonstrated significant potential for additional oil recovery, with the highest incremental recovery recorded for ZnO at 31%, followed by SiO_2_ at 27% and Al_2_O_3_ at 23%, as observed during the Amott tests.
5. The results of this study may assist in defining the screening criteria for future displacement studies involving the presented nanoparticles for the region. This may be further developed to result in low-acid-concentration hybrid nano–acid fluid deployment in the field.

## Nomenclature

*S*_wi_: irreducible water saturation
CuO: copper oxide
DI: deionized water
FW: formation water
HNAF: hybrid nano–acid fluid
HPHT: high-pressure high-temperature
IFT: interfacial tension
LCA: low-concentration acid
NiO: nickel oxide
NPs: nanoparticles
OOIP: original oil in place
SW: seawater
TDS: total dissolved solid
wt %: weight percent
Al_2_O_3_: aluminum oxide or alumina
SiO_2_: silicon dioxide or silica
ZnO: zinc oxide
